# Systematic Benchmarking of a Dry Electrode EEG Prototype Against Wet Electrode EEG Systems in Electrophysiological/Cognitive Scenarios

**DOI:** 10.3390/bios16090467

**Published:** 2026-08-27

**Authors:** Boli Pan, Shuo Ding, Yingbo Geng, Jiacheng Liang, Fali Li, Gang Wang, Xirong Li, Yanbin Dong, Rihui Li

**Affiliations:** 1 Institute of Artificial Intelligence and Brain Sciences, University of Macau, Taipa, Macau SAR 999078, China; mc46617@um.edu.mo (B.P.); mc56418@umac.mo (Y.G.); mc46553@edu.um.mo (J.L.); 2 Beijing Metaneuron Technology Co., Ltd., Beijing 100027, China; dingshuo@meta-neurons.com; 3 Center for Information in BioMedicine, School of Life Science and Technology, University of Electronic Science and Technology of China, Chengdu 610054, China; fali.li@uestc.edu.cn; 4 Key Laboratory of Biomedical Information Engineering of Ministry of Education, Institute of Biomedical Engineering, School of Life Science and Technology, Xi’an Jiaotong University, Xi’an 710049, China; ggwang@xjtu.edu.cn; 5 Key Laboratory of Neuro-Informatics and Rehabilitation Engineering of Ministry of Civil Affairs, Xi’an 710049, China; 6 Shandong Mental Health Center, Shandong University, Jinan 250014, China; shandongren82@163.com; 7 Department of Electrical and Computer Engineering, Faculty of Science and Technology, University of Macau, Taipa, Macau SAR 999078, China

**Keywords:** wet electrodes, dry electrodes, electrophysiology, simultaneous recording, resting-state EEG, single-trial classification

## Abstract

Objective: Recent advances in dry electrode EEG have enabled rapid setup and recording in unconventional scenarios. However, past developments were primarily driven by brain–computer interfaces (BCI), leaving their comparability to wet electrodes in clinical and daily life applications an open question. Here, we developed a new dry EEG system and systematically benchmarked its performance against a commercial wet EEG system across various tasks. Methods: Participants (*n* = 19) underwent simultaneous recording using both devices. We first collected resting-state EEG under both eyes-closed and eyes-open conditions, followed by a steady-state visual evoked potential (SSVEP) task at different flicker frequencies and a motor imagery (MI) task. System performance was evaluated using power spectral density (PSD), signal to noise ratio (SNR), event-related spectral perturbation (ERSP), and single-trial classification accuracy. Results: The two systems performed similarly across different tasks. During the resting state, no statistically significant differences were observed between the two systems in the PSD of the five frequency bands (*p* > 0.05 in all cases). Similarly, SNR in the SSVEP task showed no significant differences at 8 Hz, 10 Hz, and 12 Hz after correction. For cognitive tasks, classification accuracies were comparable (SSVEP: dry 80.08% ± 7.1% vs. wet 81.10% ± 6.5%; MI: dry 72.46% ± 3.89% vs. wet 70.7% ± 2.37%). Conclusions: The developed dry EEG system can effectively record electrophysiological measurements commonly employed in research and clinical settings, with quality comparable to that of traditional wet EEG systems.

## 1. Introduction

Electroencephalography (EEG) exhibits prominent advantages, including high temporal resolution, relatively low cost, and facile maintenance. The quality of EEG recordings is critically dependent on the interface integrity between the amplifier input and the skin surface [1,2]. In EEG recordings, the application of conductive gel to achieve low impedance with wet electrodes remains the gold standard. However, wet electrode EEG recording entails sequential procedural steps, including skin preparation, conductive gel application, impedance optimization, and post-recording cleanup [3]. These steps are not only time-intensive but also may elicit discomfort in subjects. During prolonged data acquisition, the conductive gel undergoes gradual desiccation, which results in a continuous degradation of its conductive properties. Furthermore, the utilization of large volumes of electrolytes to expedite impedance reduction may trigger inter-electrode bridging, particularly in dense electrode arrays [1,2,4,5]. Owing to these inherent limitations, the setup and data acquisition of wet electrode EEG typically require operation by professionally trained technicians and are constrained within a limited timeframe [6].

In contrast, dry electrodes can be directly applied to the skin surface without the requirement for conductive gel and eliminate the need for operation by trained technicians. This not only reduces both time consumption and associated costs but also enhances subjects’ comfort during the recording process [3,6]. The inherent advantages of dry electrodes have thus driven substantial technological advancements in this field in recent years. Currently, several commercially available dry EEG systems employ sensors constructed with metal pins [3,7,8]. Meanwhile, alternative designs, such as silicon-based sensors, have also been proposed and summarized in recent review studies focusing on dry electrode EEG systems [9,10]. However, compared to traditional wet electrodes, dry electrodes are often accompanied by higher impedance.

However, most prior developments in dry electrode EEG systems have been driven by the demands of brain–computer interface (BCI) applications [8,9], where design priorities typically include high interaction rates and support for short-term experimental or interactive control tasks [10,11,12,13]. In contrast, clinical diagnostics require higher signal fidelity, greater repeatability, and compatibility with standardized electrode locations, making many BCI-oriented designs difficult to integrate into medical workflows. Dry electrode EEG systems intended for clinical and real-world use must instead prioritize stability and usability, which includes reliably capturing pathological signals in hospital settings, enabling users to independently and efficiently apply the device during at-home monitoring, and maintaining long-duration, low-noise, and comfortable performance for extended or overnight recordings, such as sleep studies [3,6,14,15]. In addition, traditional comparisons typically rely on data collected in separate sessions, requiring participants to repeat the same tasks under different acquisition conditions [3,6,16]. This design unavoidably introduces confounding effects related to attention shifts, fatigue, learning, and subtle changes in posture or movement.

To bridge these gaps, we developed a novel dry electrode EEG system suitable for clinical and routine use. Through a custom EEG cap that enables truly simultaneous recording from both systems, we conducted a systematic comparison between this advanced dry electrode system and a standard wet electrode EEG system, evaluating their data quality and electrophysiological measurement performance. For each participant, the experimental protocol consisted of sequential recording sessions in which resting-state EEG was collected under eyes-closed and eyes-open conditions, followed by recordings during a steady-state visual evoked potential (SSVEP) task and a motor imagery (MI) task. Specifically, the power spectral density (PSD) of the resting EEG data is not influenced by external task demands [3,6,16]. The SSVEP tasks were implemented to evaluate the signal-to-noise ratio (SNR) and the multivariate patterns embedded in the evoked neural data [6,17]. The MI tasks were designed to elicit distinct event-related desynchronization (ERD) and event-related synchronization (ERS) patterns [18]. Classically, the core physiological marker during MI is ERD, which is represented as prominent blue power suppression regions on event-related spectral perturbation (ERSP) maps over the contralateral hemisphere. We deemed that these selected metrics were appropriate for comprehensively comparing the performance of the dry and wet EEG systems.

## 2. Methods

### 2.1. Development of a Dry EEG System

**Figure 1:**
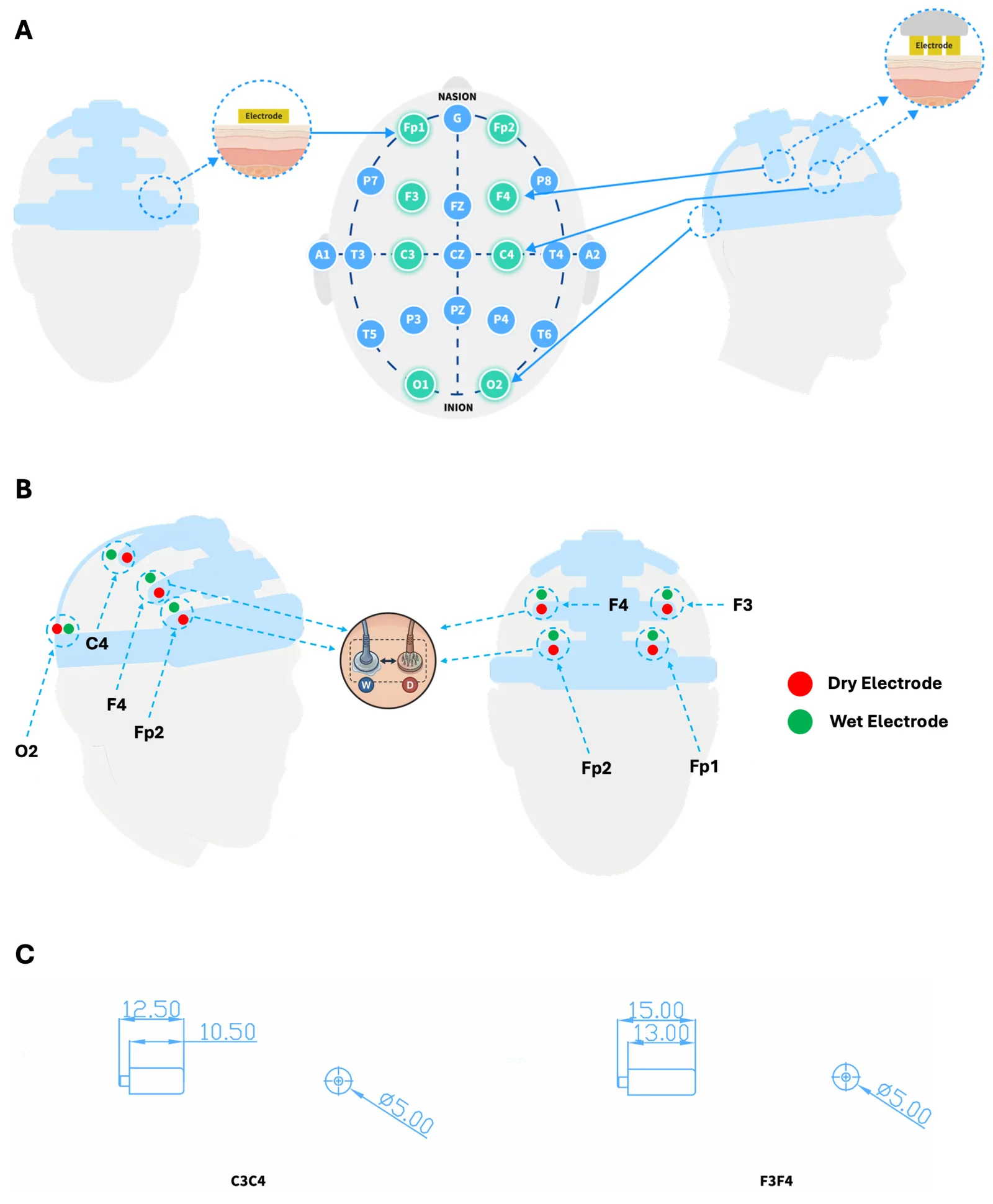
The illustration of the overall structure of the developed dry electrode EEG system and the demonstration of concurrent EEG recording using dry and wet electrode systems. (**A**) The structure of the developed dry electrode EEG system, including electrode placement, wearing configuration, and skin contact mechanism. The central diagram shows the electrode layout based on the international 10–20 system, with the eight electrodes used in this system (FP1, FP2, F3, F4, C3, C4, O1, and O2) highlighted. The head silhouettes on both sides depict how the system is positioned on the scalp during use, with the electrode modules secured by the supporting structure. The enlarged cross-sectional views show how the electrodes pass through hair and establish stable contact with the skin surface. (**B**) The demonstration of concurrent EEG recording. W, wet electrode. D, dry electrode. (**C**) The detailed structure of the dry electrodes. Left side: channel C3/C4 electrode structure. Right side: channel F3/F4 electrode structure. Unit: millimeters.

The developed dry electrode EEG system includes a novel electrode configuration (Figure 1A). Eight electrodes, including FP1, FP2, F3, F4, C3, C4, O1, and O2, are arranged according to the international 10–20 system, and this configuration supports sleep health monitoring by capturing key neural rhythms across frontal, central, and occipital regions. The frontal electrodes help detect slow wave activity and the transition from wakefulness to sleep [19,20], while the central position provides clear signals for identifying sleep spindles, K complexes, and sleep stage transitions [21]. The occipital electrodes capture alpha rhythms that indicate wakefulness and sleep onset [22]. The major novelty of the developed dry EEG system can be summarized as follows:

(1) The dry electrode consists of a nickel–titanium shape memory alloy elastic base and a stainless steel contact tip with multilayer metal coating, including a nickel transition layer and a gold outer layer. This design enhances interfacial conductivity, corrosion resistance, and contact impedance stability, with rounded edges to minimize mechanical irritation to the scalp.
(2) The geometry of the electrodes is customized based on scalp curvature, hair coverage, and pressure characteristics. Specifically, the channel F has a diameter of 5 mm and a height of 15 mm, while the channel C has a diameter of 5 mm and a height of 12.5 mm. The F3/F4 and C3/C4 electrodes are mounted at the ends of four cantilever arms, each with a specific angle to ensure a close fit to the head’s curvature (Figure 1C).
(3) The cantilever arms are fixed to the headband frame and are extendable/retractable via a knob mechanism. With adjustable ranges of 75–105 mm for region F and 95–125 mm for region C, the length can be adapted to different head circumferences, enabling precise control of electrode–scalp contact pressure, thereby ensuring stable signal acquisition and user comfort.
(4) The system is equipped with a standalone data recording unit that enables data acquisition without reliance on external tools.
(5) The system features a user-friendly interface and an easy-to-wear design, allowing individuals without professional training to operate it.

### 2.2. Participants

A total of 19 college students (25.32 ± 4.11 years) were recruited from the University of Macau (11 females and 8 males). All participants had normal or corrected vision and reported no neurological or physical health issues, nor did they experience poor sleep quality in the past week. They signed the informed consent form electronically. The study was approved by the RESEARCH COMMITTEE - PANEL ON RESEARCH ETHICS of the University of Macau (BSERE23-APP012-ICI).

### 2.3. Concurrent EEG Recording Using Dry and Wet Electrode Systems

During electrode placement and EEG recording, participants sat comfortably at 60 cm from the screen. The wet electrode EEG data were collected using equipment manufactured by Biosemi (Amsterdam, The Netherlands). Previous studies demonstrated that the power and waveform of the scalp EEG remain highly stable within a 10 mm displacement [23,24]. Therefore, the channels of the wet electrode system and the dry electrode system are each 4 mm away from the standard positions of the international 10–20 system, as shown in Figure 1B. The mastoid electrodes of the two recording systems are slightly different in position. The mastoid electrodes of the dry electrode system are located 5 mm above those of the wet electrode system. Before each recording session, we checked the electrode impedance and signal quality of both the dry and wet electrodes to ensure proper system operation and correct placement. After data acquisition, both systems were cleaned and maintained to ensure consistent device performance across all participants.

### 2.4. Experimental Design

#### 2.4.1. Resting-State EEG Recording

**Figure 2:**
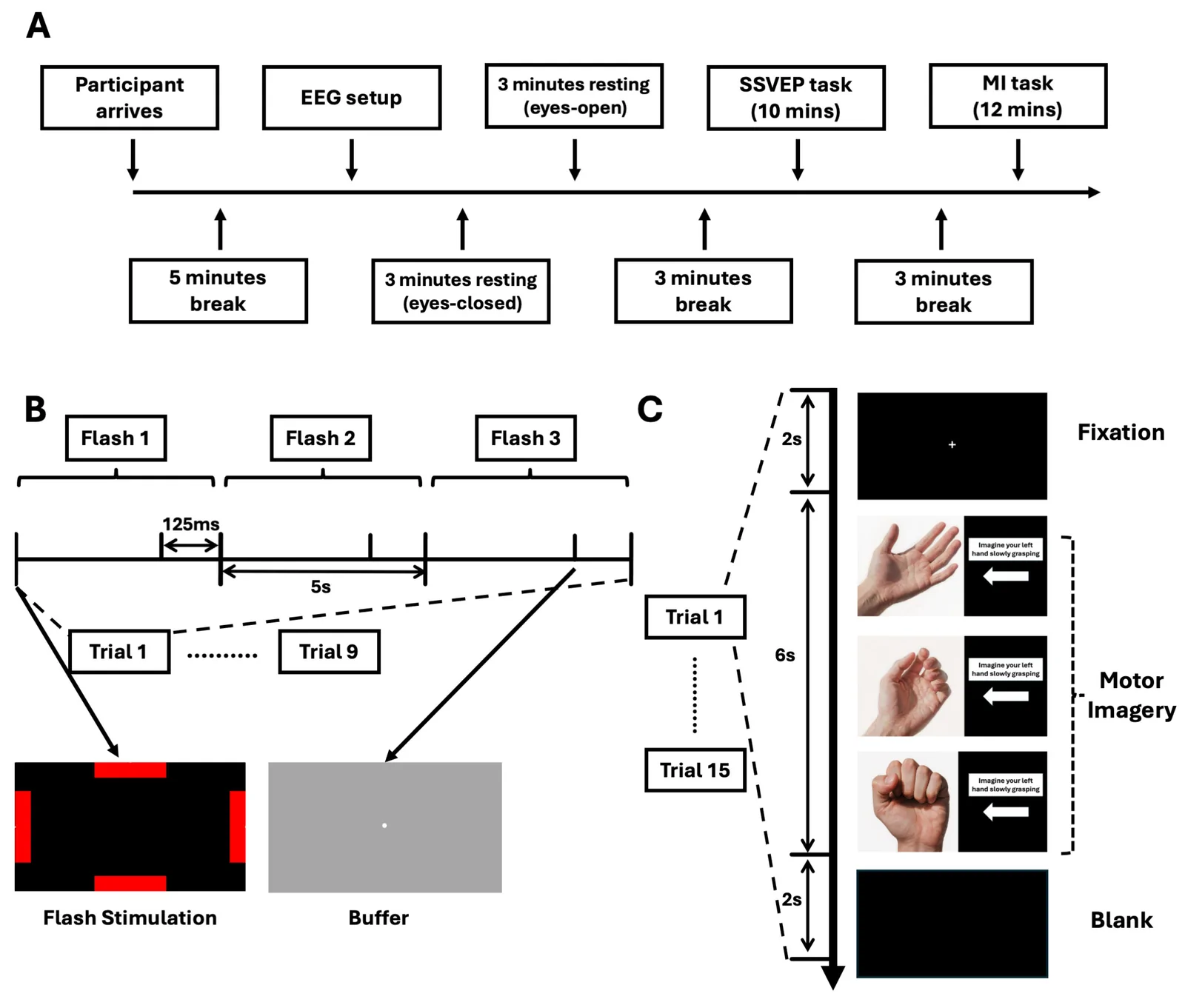
The task paradigms. (**A**) The flowchart of the whole experiment. (**B**) Steady-state visual evoked potential (SSVEP) task. (**C**) Motor imagery (MI) task.

Two resting-state EEG recordings were collected (Figure 2A). The first experimental task consisted of a 3-min resting EEG recording with eyes closed, during which participants were instructed to remain relaxed and minimize movement. The second experimental task involved another 3-min resting EEG recording with eyes open, allowing assessment of EEG activity under a contrasting state of visual engagement and alertness.

#### 2.4.2. Steady-State Visual Evoked Potential Task

In the third experimental task, participants completed a standard SSVEP paradigm (Figure 2B). They were instructed to direct their attention toward a designated flickering target throughout each stimulation period. Each flicker sequence lasted 4.875 s and was followed by a 125 ms interval before the next sequence began [17,25,26]. Three such flickers formed one trial, and each block contained nine trials. A 1-min resting period was provided after every 2 consecutive blocks to minimize fatigue, and there were a total of 4 blocks in this task.

#### 2.4.3. Motor Imagery Task

The fourth experimental task employed a traditional MI paradigm [18,27,28]. The task involved imagining a left-hand and a right-hand grasping condition (Figure 2C). Before each trial, a fixation point appeared on the screen for 2 s, followed by a sequence of three artificially generated images of left-hand and right-hand grasping displayed over the next 6 s, with a 2-s blank appearing afterward. Each image was accompanied by directional arrows and corresponding characters. Participants were instructed to focus on the MI of the movements rather than their visual perception. Each block contained 15 trials, and the left-hand and right-hand grasping conditions were performed alternately. There was a 1-min resting period between every 2 blocks.

### 2.5. EEG Data Processing and Quantification

#### 2.5.1. Data Preprocessing

EEG data from two EEG systems were preprocessed using an identical pipeline implemented in EEGLAB (v2025.0.0) [29]. The continuous datasets were bandpass filtered between 1 and 40 Hz, and offline rereferenced to the average of the two mastoid electrodes [30]. Independent component analysis (ICA) was subsequently applied to identify and remove components associated with ocular and muscle artifacts.

#### 2.5.2. Resting-State EEG Data Analysis

Continuous EEG data were divided into non-overlapping 2 s epochs. Spectral analysis was performed for each epoch using fast Fourier transform (FFT) with a Hanning window, from 1 Hz to 40 Hz in 1 Hz steps [3,6,30]. The PSD for each participant was then averaged across all epochs for each electrode. Finally, we calculated the mean PSD across five frequency bands, including delta (2–4 Hz), theta (4–8 Hz), alpha (8–14 Hz), beta (14–30 Hz), and gamma (30–40 Hz). To examine the differences in PSD of the same subject across different systems, we conducted paired t-tests for each of the five frequency bands with false discovery rate (FDR) correction (*p* = 0.05).

#### 2.5.3. SSVEP Data Analysis and Classification

PSD and SNR were computed for the SSVEP data, with channels O1 and O2 from both dry and wet electrode systems [6,11,17]. PSD was estimated using Welch’s method [31,32]. Epoch length was set to 6 s with 50% overlap to minimize spectral leakage. A Hamming window was applied to each epoch to reduce edge effects, resulting in a frequency resolution of 0.5 Hz. PSD values were computed across the 5–40 Hz frequency range and subsequently normalized to mitigate the influence of outliers [6,11]. The PSD was calculated as follows:

(1) $$P_{xx}\left(f\right)=\frac{1}{U\cdot f_{s}}\cdot \frac{1}{K}\sum_{i=0}^{K-1}{\left|x_{i}\left(f\right)\right|}^{2}$$

where *K* denotes the number of segments, *X_i_* (*f*) represents the discrete Fourier transform of the *i*-th windowed segment, *U* is the normalization factor of the window function, and *f_s_* stands for the sampling frequency. The *SNR* was calculated as follows:

(2) $$SNR=10\log_{10}\left(\frac{P_{signal}}{P_{noise}}\right)\left(dB\right)$$

To examine the differences in *SNR* across different systems, we conducted paired t-tests with FDR correction.

In addition, we applied a random forest-based classification approach to evaluate the performance of the dry and wet electrode EEG systems in distinguishing between 3 different SSVEP conditions [33,34,35,36]. The features included FFT-based PSD estimates at the target stimulation frequencies and their harmonics, power ratios, band power ratios across standard frequency bands, and time domain statistics such as the mean, standard deviation, skewness, and kurtosis [37]. The resulting feature matrix was normalized within each fold of cross-validation. Classification was trained with 200 trees and curvature-based predictor selection. Model performance was evaluated using five-fold cross-validation. For each fold, the model was trained on the training subset, and predictions as well as class probability scores were generated for the held-out samples. Out-of-fold probability estimates were stored for every epoch, enabling unbiased computation of multiclass one-vs-rest area under the curve (AUC) values. Classification performance was separately evaluated for dry and wet electrodes using accuracy, sensitivity, specificity, precision, F1 score, and AUC computed from the receiver operating characteristic (ROC).

#### 2.5.4. Motor Imagery Data Analysis and Classification

The ERSP allows us to observe the spectral power changes of the MI-induced EEG from the views of the time frequency domain [18,38]. ERSP values were calculated from −500 ms to 6500 ms based on electrode positions C3 and C4, and mean ERSP values were displayed between 5 and 35 Hz after normalization. The *ERSP* was calculated as follows:

(3) $$ERSP\left(f,t\right)=\frac{1}{n}\sum_{k=1}^{n}F_{k}{\left(f,t\right)}^{2}$$

where *n* is the number of trails and *F_k_* (*f*, *t*) is the spectral estimation of the *k*-th trial at frequency *f* and time *t*. The common spatial pattern (CSP) aims at learning spatial filters that maximize the variance of bandpass-filtered EEG signals from one class while minimizing their variance from the other class [39,40,41,42]. Formally, CSP uses the spatial filters *w* that extremize by the following function:

(4) $$J\left(w\right)=\frac{w^{T}X_{1}^{T}X_{1}w}{w^{T}X_{2}^{T}X_{2}w}=\frac{w^{T}C_{1}w}{w^{T}C_{2}w}\;$$

where *T* denotes transpose, *X_i_* is the data matrix for class *i* (with the training samples as rows and the channels as columns), and *C_i_* is the spatial covariance matrix from class *i*.

Similarly, we applied a random forest framework to evaluate the performance of the dry and wet electrode EEG systems in distinguishing between 2 different MI conditions [43,44]. For each trial, a comprehensive feature set was extracted, including CSP and PSD for every trial. Time domain characteristics were also calculated, including the mean, standard deviation, skewness, and kurtosis. Model selection was performed using a stratified five-fold cross-validation scheme [45]. A grid search over the number of trees, minimum leaf size, and number of predictors sampled per split was executed, and the configuration yielding the highest mean validation accuracy was selected as the optimal parameter set. Following parameter selection, a final evaluation was conducted with the best configuration using a new five-fold cross-validation run. Classification performance was separately evaluated for dry and wet electrodes using accuracy, sensitivity, specificity, precision, F1 score, and AUC computed from the ROC.

## 3. Result

### 3.1. Resting-State EEG with Eyes Closed and Open

**Figure 3:**
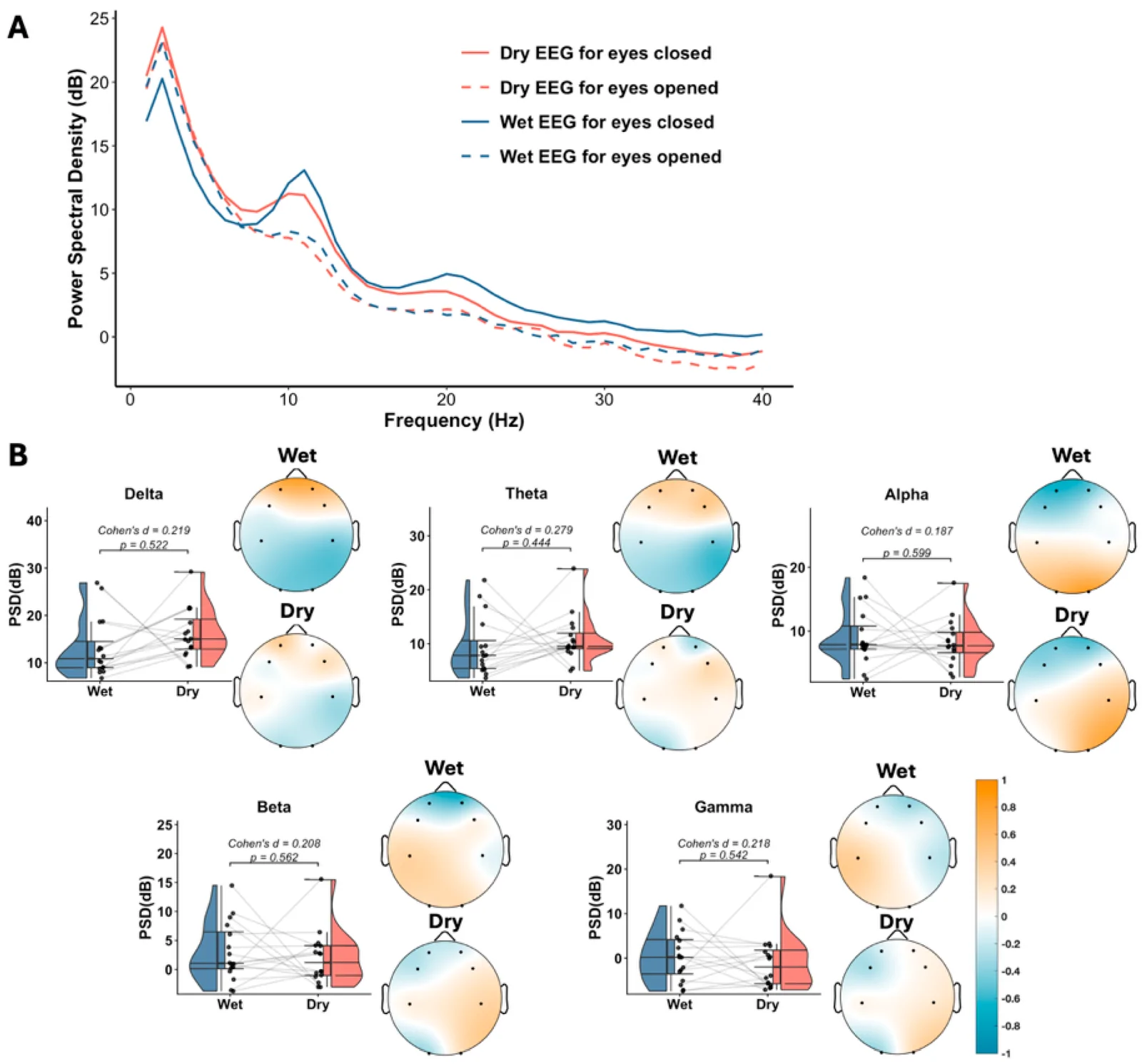

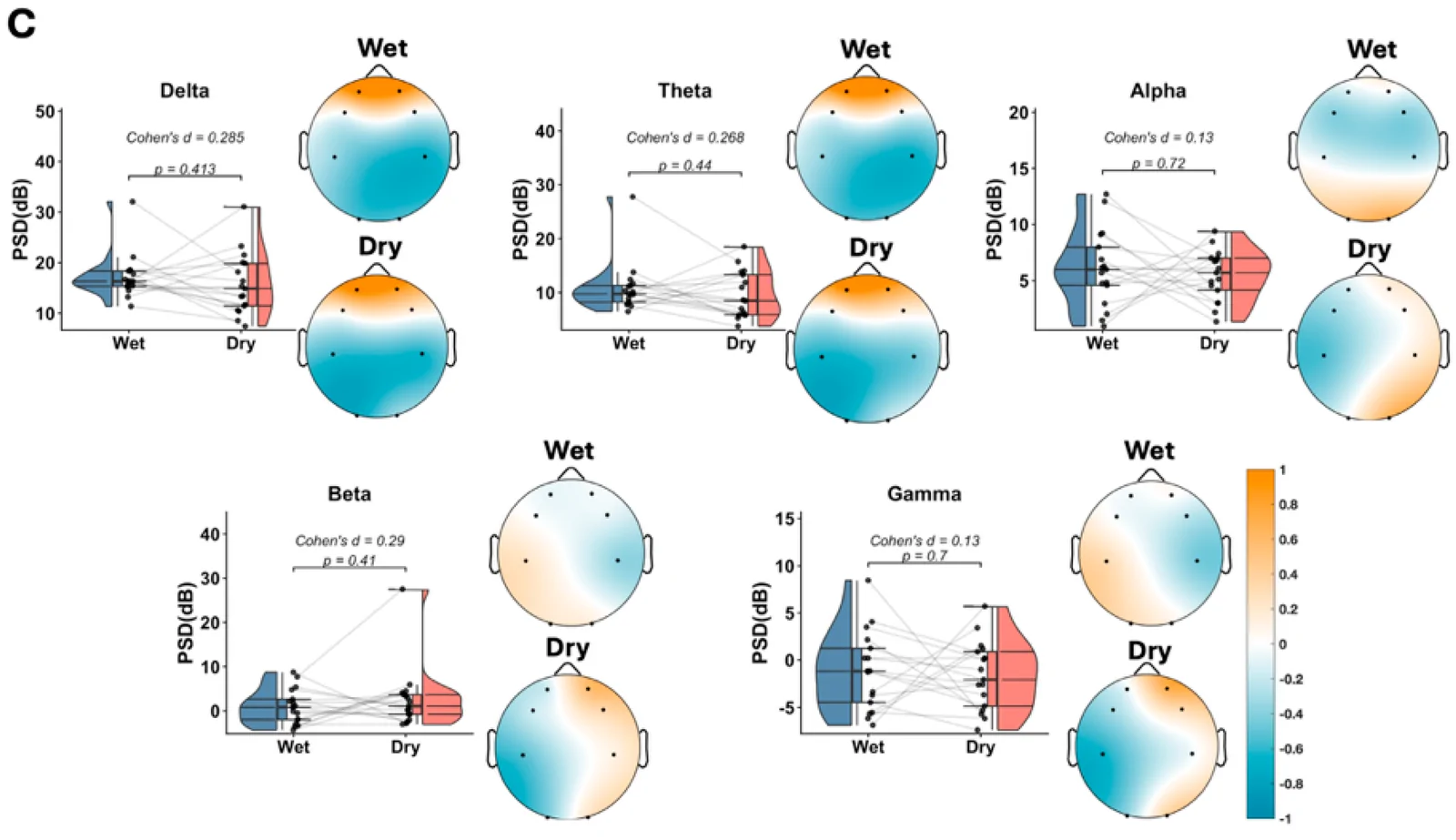
The power spectrum density (PSD) and topography analysis of resting-state EEG data. (**A**) The PSD averaged across all electrodes for dry and wet electrode systems is shown separately for eyes open and closed. (**B**) The scatterplots of mean PSD for delta, theta, alpha, beta, and gamma during the eyes-closed condition for the wet and dry EEG systems. *p*-Value and Cohen’s *d* of the paired t-test were reported for each scatterplot. Topographic plots were also plotted separately for each frequency band and EEG system. (**C**) The same scatterplot and scalp topography were plotted for the eyes-open condition. The color bar indicates z-normalized PSD from −1 to 1.

Figure 3 illustrates the PSD across all the electrodes, the topography of each frequency band, as well as the distribution of PSD collected by two systems for the same subject. The average PSD across all electrodes was comparable between the two systems throughout the entire frequency range examined. For the two systems, the PSD of the alpha wave was consistently higher in the eyes-closed state compared to the eyes-open condition (Figure 3A). The mean PSD for each system, as well as the *p*-values, 95% confidence intervals (CIs), and Cohen’s *d* comparing the two systems for each frequency band and for both resting-state conditions, are reported in Table 1. In the eyes-closed condition, no frequency bands showed differences between systems. Similar results were observed in the eyes-open condition.

### 3.2. Steady-State Visual Evoked Potential Paradigm

**Figure 4:**
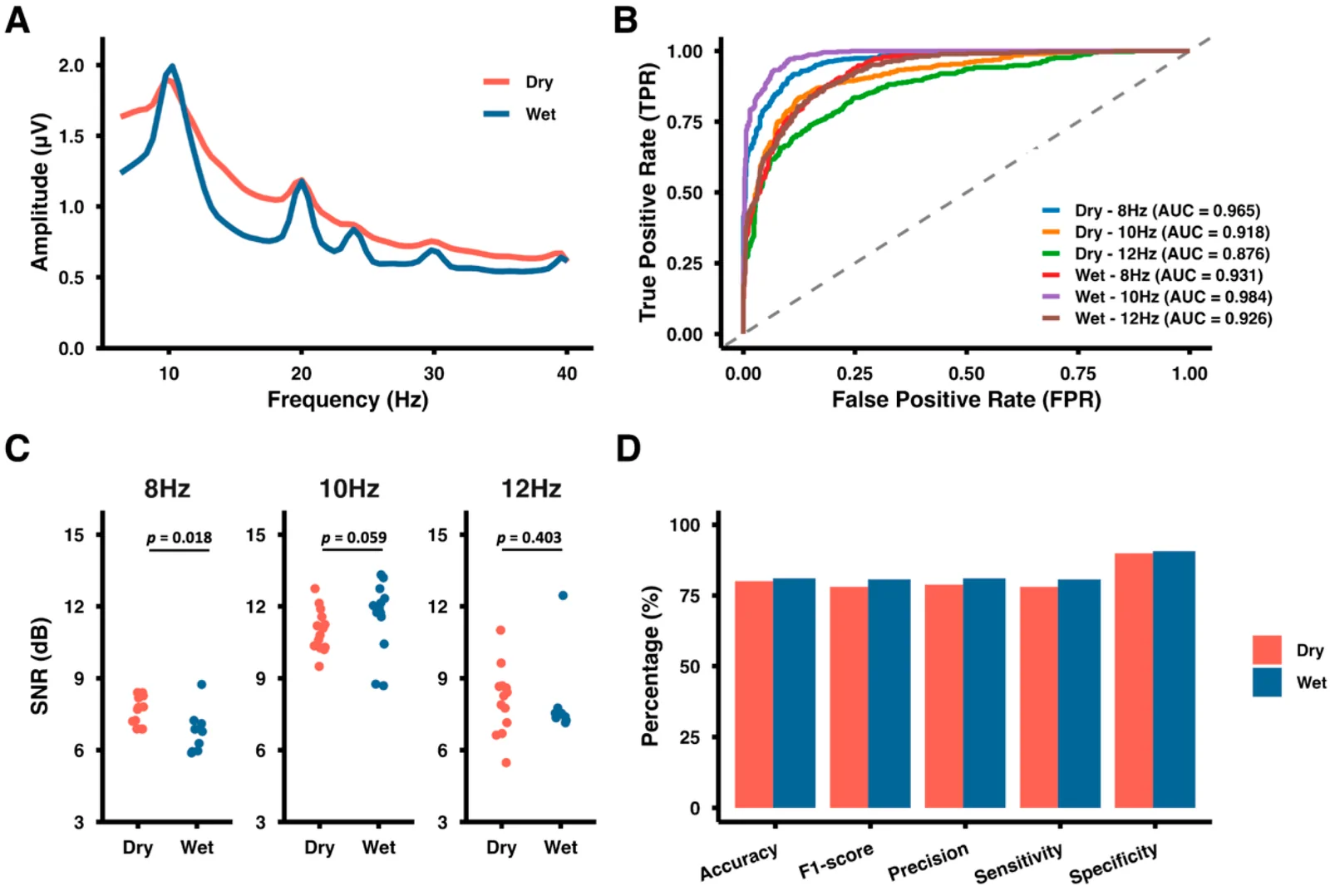
Steady-state visual evoked potential (SSVEP) amplitude spectrum, signal to noise ratio (SNR), and the performance of classification. (**A**) The averaged amplitude spectrum at 10 Hz after normalization. Dry electrode system (red line) and wet electrode system (blue line). (**B**) The ROC (receiver operating characteristic curve) and AUC (area under the curve) of classification. (**C**) Averaged SNR of dry and wet electrode systems in different flicker frequencies (8 Hz, 10 Hz, and 12 Hz). (**D**) The parameters that were evaluated for the performance of the classification.

Figure 4A illustrates the PSD amplitude of SSVEP recorded from the subject using both dry and wet electrode systems. Both systems effectively captured clear SSVEP, with a prominent peak frequency at 10 Hz, along with harmonic frequencies at 20 Hz, 25 Hz, and 30 Hz. There were no significant differences in SNR between the dry and wet electrode systems at 8 Hz (7.64 ± 0.60 vs. 6.76 ± 0.91, *p* = 0.018, 95%CI = [0.126, 1.630]), 10 Hz (dry: 10.94 ± 0.87; wet: 11.58 ± 1.47, *p* = 0.059, 95% CI = [−1.614, 0.333]), or 12 Hz (dry: 8.06 ± 1.41; wet: 8 ± 1.68, *p* = 0.403, 95% CI = [−1.395, 1.514]) after FDR correction, as illustrated in Figure 4C.

### 3.3. The Classification Performance of the Steady-State Visual Evoked Potential Paradigm Dataset from Dry and Wet Electrode Systems

Classification performance was evaluated for both the dry and wet electrode systems across five metrics, and the two systems exhibited comparable performance. The dry electrode system achieved an accuracy of 80.08 ± 7.1%, a sensitivity of 78.06%, a specificity of 89.92%, a precision of 78.86%, and an F1 score of 78.02%. The wet electrode system showed slightly higher performance, with an accuracy of 81.10 ± 6.5%, a sensitivity of 80.70%, a specificity of 90.63%, a precision of 80.97%, and an F1 score of 80.78% (Figure 4D). For the dry electrode system, AUC values were 0.965 at 8 Hz, 0.918 at 10 Hz, and 0.876 at 12 Hz. For the wet electrode system, the corresponding AUC values were 0.931, 0.984, and 0.926, as illustrated in Figure 4B.

### 3.4. Motor Imagery Paradigm

**Figure 5:**
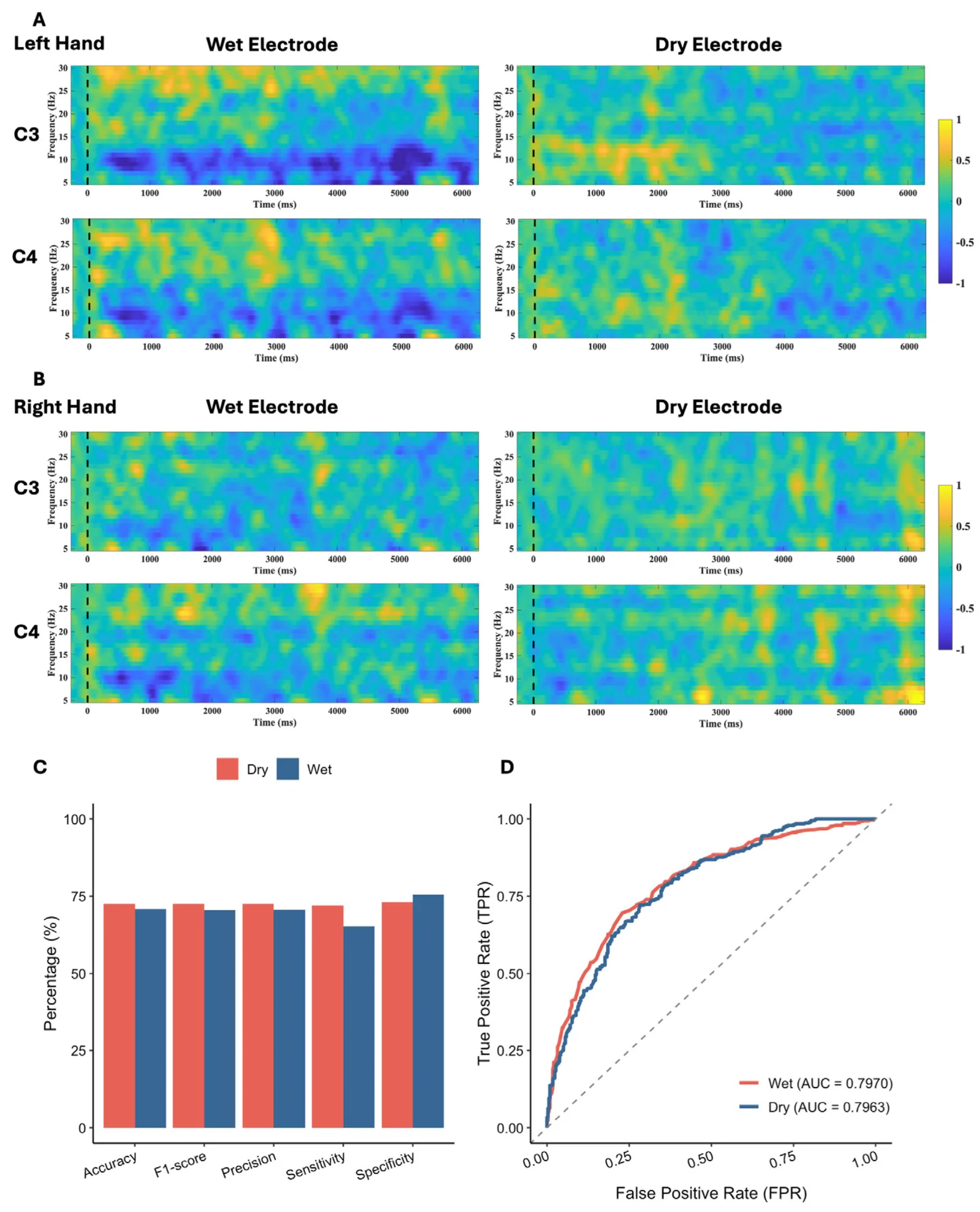
Group-averaged time frequency map of dry and wet electrode systems of channels C3 and C4, and the performance of classification. (**A**) Left-hand grasping condition. (**B**) Right-hand grasping condition. The color bar adjacent to the time frequency map represents the power after normalization. (**C**) The classification performance of the dry and wet systems. (**D**) The ROC (receiver operating characteristic curve) and AUC (area under the curve) of classification.

Figure 5 presents the time frequency plots for all subjects during the two MI tasks at electrode positions C3 and C4. Across all MI conditions, a sustained decrease in power was observed. In the left-hand grasping condition (Figure 5A), the wet electrode system showed a stronger and more sustained ERD pattern. Notably, under this condition, the dry electrode system did not exhibit a significant ERD pattern at the onset of stimulation. In the right-hand grasping condition (Figure 5B), both the C4 channels of the dry and wet electrode systems displayed a similar pattern. However, the wet electrode at C3 also showed a stronger and more sustained ERD pattern.

### 3.5. The Classification Performance of Motor Imagery Dataset from Dry and Wet Electrode Systems

Classification performance for MI was assessed using five metrics for both dry and wet electrode systems, and the two systems exhibited close performance. For the dry electrode system, the accuracy was 72.46 ± 3.89%, the sensitivity was 71.88%, the specificity was 73.04%, the precision was 72.47%, and the F1 score was 72.46%. For the wet electrode system, the accuracy was 70.7 ± 2.37%, the sensitivity was 65.29%, the specificity was 75.45%, the precision was 70.63%, and the F1 score was 70.43% (Figure 5C). The area under the ROC curve showed comparable performance across electrode types, with an AUC of 0.7963 for the dry electrode system and 0.7970 for the wet electrode system, as illustrated in Figure 5D.

## 4. Discussion

In this study, we conducted a comprehensive systematic benchmarking of a novel dry electrode EEG system against a commercially established wet electrode EEG system, evaluating their signal performance across a suite of experimental paradigms and quantitative electrophysiological metrics. The two systems exhibited highly comparable performance across all assessed tasks, with the dry electrode system effectively capturing key characteristics of EEG data, including frequency band power, event-related spectral dynamics, and multivariate neural activity patterns. Particularly in resting-state EEG recordings, this dry electrode EEG system, which is designed primarily for sleep monitoring, demonstrates performance comparable to that of traditional wet electrode systems, highlighting its outstanding capabilities in the field of sleep monitoring. Collectively, these findings confirm that the developed dry EEG system is capable of reliably detecting the core electrophysiological indices routinely utilized in both neuroscientific research and clinical practice.

Resting-state EEG recordings revealed no statistically significant differences in spectral power between the dry and wet systems across the delta, theta, alpha, beta, and gamma frequency bands, for both eyes-closed and eyes-open conditions. For these frequency bands, the scalp topographies generated by the two EEG systems were similar, with the main differences concentrated in the beta and gamma bands under the eyes-open condition, which may be related to the slight differences in electrode placement between the two systems. Resting-state EEG recordings reflect ongoing neural processes that occur in the absence of external task involvement [3,6], including those during wakefulness and sleep [14,32]. In real-world applications, such metrics have become particularly valuable in the domain of sleep monitoring, where spontaneous EEG rhythms provide reliable indicators of sleep architecture, transitions between wakefulness and sleep, and fluctuations in circadian regulation. Power spectral features across delta, theta, alpha, and sigma bands are routinely employed to identify sleep stages, detect micro-arousals, assess sleep continuity, and quantify sleep quality under both clinical polysomnography and home-based monitoring frameworks. Moreover, resting-state EEG signatures have demonstrated substantial discriminative power in distinguishing healthy sleep from pathological sleep phenotypes. Altered power distributions in slow wave and spindle-related frequencies have been associated with conditions such as insomnia, sleep apnea, neurodegenerative disorders, and mood disorders [46,47]. The findings of our study thus support the feasibility of using dry EEG systems for continuous or home-based sleep assessment, providing a robust and accessible alternative to traditional wet electrode systems. Additionally, differentiation based on the texture features of dry and wet eyes may be possible [48].

For the SSVEP task, both systems successfully captured distinct SSVEP signals at occipital channels O1 and O2, as evidenced by the clear fundamental and harmonic frequency peaks, validating the rationality of the dry electrode system’s channel layout and sensor design for visual evoked potential recording. In particular, we found that there were no significant differences in SNR between the two systems at 8 Hz, 10 Hz, and 12 Hz after correction, demonstrating the dry system’s robust signal detection capability for visual cortical responses. However, there was a slight difference in the amplitude of data acquired by the dry electrode system compared with the wet electrode system, which may be attributed to the lower impedance of the wet electrode system. In addition, we observed that data acquired solely using the dry electrode system contained fewer artifacts and exhibited lower impedance. When both systems were used simultaneously, the EEG paste from the wet electrodes may have impaired the dry electrodes’ sensing performance. Dry electrodes are highly sensitive to skin oils, moisture, and gel residue, and the presence of EEG gel can alter circuit pathways, increasing motion artifacts and low-frequency drift [49,50]. Despite these minor technical confounds, the dry and wet systems yielded comparable classification performance for SSVEP data, confirming the dry system’s strong utility for visual cognitive tasks [51,52]. SSVEP has been shown in research to reflect color perception and attention mechanisms, and in clinical settings it has been used as a marker for mild cognitive impairment and schizophrenia [53,54]. Given the widespread use of the SSVEP and the advantages of dry electrodes, dry electrode EEG systems can be conveniently deployed across large participant samples in more naturalistic environments, including patients’ homes or clinics.

In the MI task, the wet electrode system exhibited more pronounced and sustained ERD in the alpha and beta bands during motor imagery, a difference we attribute to two key experimental confounds: interference from the wet system’s conductive gel on the dry electrode–skin interface, and small positional differences in the mastoid reference electrodes between the two systems [49,50]. Prior research has established that even minor shifts in reference electrode placement can alter the amplitude and temporal dynamics of event-related brain potentials [55]. However, the dry electrode system even achieved higher classification accuracy, suggesting that the dry system reliably captures the multivariate neural features that encode motor intention, confirming its acceptable performance for MI-based paradigms. In clinical applications, the gel-free setting of dry electrode EEG systems is vital for the reliable detection of patients’ motor intentions without the need for conductive gel or complex preparation [1,2,3]. The reliable performance of our proposed dry electrode EEG in the MI task makes it well suited for home-based neurorehabilitation, long-term motor function monitoring, and assistive BCI systems for patients with motor impairments such as stroke, spinal cord injury, or neurodegenerative diseases [56,57,58].

The promising performance of the developed dry EEG system may be attributed to several key design features. In previously reported dry EEG systems [3,5,6], the primary focus has been on the development of dry electrodes themselves, while data acquisition typically relies on separate recording hardware, external computers, and dedicated software. In contrast, the proposed system incorporates the developed dry electrodes into a dedicated EEG cap specifically designed to improve wearing comfort during prolonged recordings, such as overnight sleep monitoring. The cap structure also simplifies electrode positioning, allowing users with minimal training to operate the system independently without relying on professional assistance. Furthermore, the proposed system integrates the EEG cap, portable data-recording unit, and a user-friendly software interface into a single platform. This integrated design reduces system complexity, eliminates the need for external acquisition equipment, and enhances portability as well as ease of use. Together, these features enable practical long-term EEG monitoring in real-world settings while improving user autonomy and accessibility.

A key strength of the present study is the use of simultaneous co-recording with both dry and wet electrode systems, which eliminates confounding variables inherent to separate-session comparisons [59]. However, this experimental design also introduced notable limitations that warrant acknowledgment. First, simultaneous wear of both systems precluded an accurate quantification of the dry system’s practical advantages, including electrode preparation time, ease of application, and participant subjective comfort. Second, the conductive gel used for the wet electrode system may likely interfere with the dry electrode–skin interface, potentially attenuating the dry system’s performance in the co-recording setting and underestimating its standalone signal quality. Third, small positional offsets in both recording and reference electrodes between the two systems represent a minor confounding factor for the comparison of event-related spectral and potential dynamics. Admittedly, the sample size of this study remains relatively modest. Testing across larger population groups may be required to further confirm the generalization of these outcomes. It is also worth noting that the dry electrode system has so far been validated only for short-term performance under standard laboratory conditions. Its performance during prolonged use and under different real-world conditions warrants further investigation.

Overall, the developed dry electrode EEG system demonstrated signal quality and electrophysiological detection performance highly comparable to the gold-standard wet electrode system across resting-state, SSVEP, and MI paradigms, three core experimental approaches in neuroscientific research. These findings provide empirical support for the dry electrode system as a promising alternative to traditional wet EEG setups. While future studies are required to directly evaluate real-world translation and prolonged usage, the system’s ease of use and minimal preparation requirements suggest favorable potential for future applications in home-based monitoring, population studies, and neurorehabilitation interventions.

## Figures and Tables

**Table 1:** Descriptive and statistical values for PSD at resting state EEG. The PSD for the wet and dry system, as well as the *p*-values and Cohen’s *d* for each frequency band comparing the two systems, are reported for the resting state with eyes open and closed. CI: confidence interval.

Frequency	WET	DRY	*p*	95% CI	Cohen’s *d*
Eyes-Closed					
Delta	12.95	16.16	0.522	[−2.124, 8.105]	0.219
Theta	9.44	10.85	0.444	[−3.301, 6.306]	0.279
Alpha	9.02	8.26	0.599	[−4.487, 2.982]	0.187
Beta	2.50	1.87	0.562	[−5.178, 3.147]	0.208
Gamma	0.82	−0.32	0.542	[−6.163, 3.514]	0.218
Eyes-Open					
Delta	15.46	17.04	0.413	[−2.217, 5.242]	0.285
Theta	9.63	10.76	0.44	[−1.656, 4.078]	0.268
Alpha	6.07	6.53	0.72	[−2.158, 3.242]	0.13
Beta	0.83	2.96	0.41	[−2.071, 5.311]	0.29
Gamma	−1.15	−1.67	0.7	[−3.744, 2.703]	0.13

## Data Availability

The data presented in this study are available on request from the corresponding author due to commercial confidentiality restrictions and the ongoing patent process of the newly developed device.
